# Epidemiologic characteristics and double-buffered strategy for patients in orthopedic surgery during the novel coronavirus outbreak: Wuhan’s experience

**DOI:** 10.1186/s40001-021-00510-0

**Published:** 2021-04-30

**Authors:** Yan Zhou, Jianghua Ming, Shiqing Liu

**Affiliations:** Department of Orthopedics, Renmin Hospital of Wuhan University, #238 Jiefang Road, Wuhan, 430060 Hubei People’s Republic of China

**Keywords:** Novel coronavirus 2019, Viral pneumonia, Fracture, Diagnosis and treatment mode, Infection control

## Abstract

**Background:**

The purpose of this article is to summarize the epidemiologic characteristics and double-buffered strategy for patients in orthopedic surgery during the COVID-19 outbreak in Wuhan, China, based on our own experience in our hospital.

**Methods:**

A retrospective and comparative study was performed to identify all inpatients at our clinic from February 17 to April 20, 2020 (epidemic group), and from February 17 to April 20, 2019 (control group). Epidemiologic characteristics, screening effect, perioperative complications, and nosocomial infection were analyzed.

**Results:**

In the epidemic group, 82 patients were identified, a decrease by 76.0% than the 342 patients in the same period in the 2019. Patients in the epidemic group (54.6 ± 20.2 years) were older than those in the control group (49.6 ± 22.5 years). For the epidemic group, the proportion rates of traumatic factures (69.5%) and low-energy injuries (86.0%) were higher than that in the control group, respectively (35.4% and 37.2%). The preoperative waiting time (7.0 ± 2.6 days) in the epidemic group was longer than that in the control group (4.5 ± 2.1 days). The postoperative complication rate (12.2%) in the epidemic group was higher than that in the control group (3.5%). No nosocomial infection of orthopedic staff and patients with COVID-19 was noted in the epidemic group.

**Conclusion:**

During the COVID-19 outbreak in Wuhan, China, orthopedic inpatients showed unique epidemiological characteristics. The double-buffered strategy could effectively avoid nosocomial infections among medical staff and patients. Doctors should fully evaluate the perioperative risks and complications.

## Background

In December 2019, the novel coronavirus disease 2019 (COVID-19) caused by severe acute respiratory syndrome coronavirus 2 (SARS-CoV-2) spread throughout Wuhan, China [1, 2]. Today, the disease has infected individuals from nearly all countries of the world, including Italy [3], the United States [4], and Australia [5]. COVID-19 is considered a category B infectious disease, as stipulated by the law of the People’s Republic of China on the prevention and control of infectious diseases, and prevention and control measures for category A infectious diseases have been adopted to control it [6].

Aerosol transmission may occur when a patient is exposed to high-concentration aerosols for long periods of time in a relatively closed environment [7]. According to a recent report, the infection rate in the hospital population is approximate 41%, of which 29% comprise hospital staff and 12.3% are inpatients [8]. The scientific prevention and control of the COVID-19 epidemic has entered a new stage in Wuhan, and asymptomatic infection of the coronavirus has become the main source of nosocomial spread. Compared with their counterparts in the field of infectious diseases, orthopedic surgeons are not usually considered front-line staff in the fight against viral pandemics. However, as part of the larger healthcare ecosystem, orthopedic surgeons also play crucial role in controlling the disease. The purpose of the present retrospective study is to analyze the clinical characteristics of patients in orthopedic surgery and identify the most appropriate management strategy for COVID-19 according to our own experience in our hospital. We compared the results with a cohort of patients treated during the same seasonal period 1 year ago with respect to epidemiological characteristics and management of patients. The results of this work may provide a reliable and accurate basis for the development of orthopedic prevention and control strategies.

## Materials and methods

### Study design and participants

This study involves a retrospective case series using data prospectively gathered from February 17 to April 20, 2020 and from February 17 to April 20, 2019 at the Orthopedic Surgery Department of our hospital. All methods were conducted following the relevant regulations and guidelines of the Institutional Review Board of the hospital.

### Data collection

We reviewed the electronic medical records, preoperative evaluation records, nursing records, anesthesia and operation records, laboratory findings, and chest computed tomography (CT) scans of all patients. After obtaining written informed consent from these patients, all clinical data were independently collected by two investigators. The cause of injury was reported by the patients, and similar cases were grouped together. All patients were divided into two groups: epidemic period group (patients admitted in 2020), and control group (patients admitted in 2019 of the same seasonal period). The primary data collected included the gender and age of patients, the indication for hospital admission and surgical treatment, type of diseases, injury causes and location, preoperative waiting time, screening effect of the double-buffered management mode, perioperative complications, and nosocomial infection. Medical records were analyzed using a customized data-collection form.

To clarify the urgency of orthopedic surgery, we have categorized orthopedic surgeries into five categories based on priority: Priority A (emergency surgery within 24 h, Priority B (urgent surgery within < 48 h, Priority C (Expedited Surgery within 2 weeks), Priority D (Short-Term Delayed < 3 months), and Priority E (Long-Term Delayed > 3 months) [9]. The categories of Priority A and B are classified as orthopedic emergency, Priority C is classified as expedited surgery, and Priority D and E are classified as elective surgery.

### Inpatient triage workflow

**Fig. 1:**
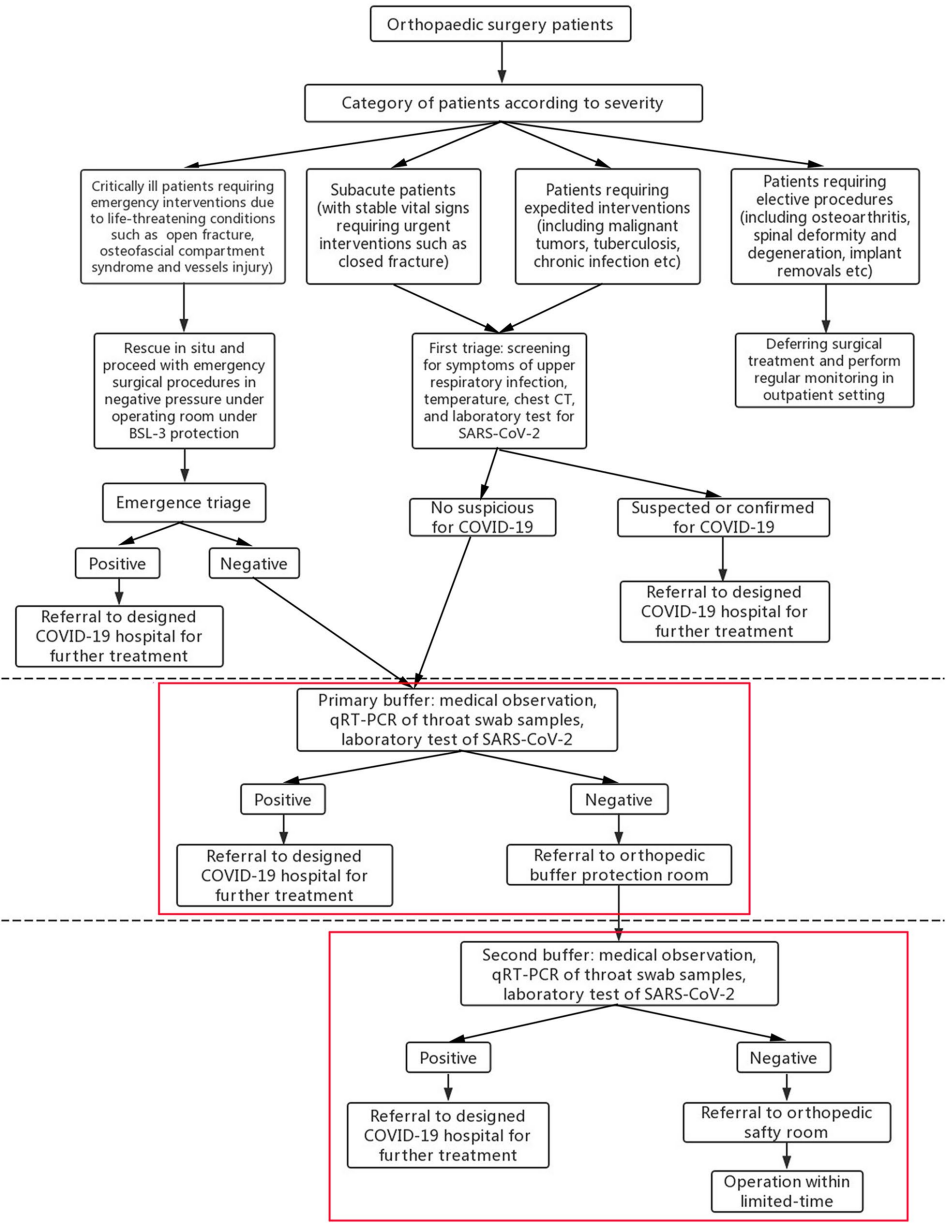
Flowchart of double-buffered diagnosis and treatment mode for orthopedic surgery patients

**Fig. 2:**
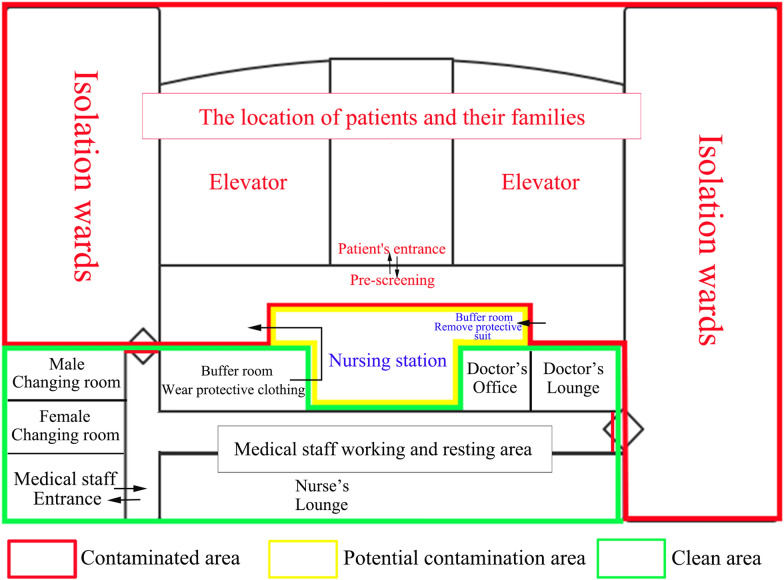
Schematic diagram of comprehensive buffer ward

Double-buffer wards were set up for management according to the characteristics of orthopedic patients during the COVID-19 outbreak; these wards were established to prevent, control, eliminate the harm caused by COVID-19 in an effective and timely manner and ensure that orderly normal medical treatment work is carried out. All patients underwent strict pre-examination and triage in the outpatient clinic, including detailed inquiries about the patient’s past and contact history, and received temperature monitoring, chest CT, and routine blood, nucleic acid and serological antibody testing. After the patient was admitted to the hospital, two buffer transitions were performed in the comprehensive buffer ward (2–3 days) and the orthopedic ward buffer room (2–3 days). During this period, the patient’s temperature and COVID-19-related symptoms were closely observed. The patient underwent routine blood, nucleic acid, and serological antibody testing once more. After excluding COVID-19, the patients were transferred to an orthopedic safe patient ward for specialist orthopedic treatment. The comprehensive buffer ward was divided into three areas, including a clean area, potentially contaminated area, and contaminated area, and two channels, including medical staff and patient aisles, for management. Figure 1 illustrates the inpatient triage workflow, and Fig. 2 illustrates the schematic diagram of comprehensive buffer ward.

### Emergency surgical strategy in patients with COVID-19

For patients with COVID-19 who required emergency interventions due to life-threatening conditions, such as open fractures, osteofascial compartment syndrome, blood vessels and nerves injury, and large area avulsion skin injuries, all healthcare providers were required to adhere to strict prevention and infection control protocol in addition to the practice of routine universal precaution. Medical personnel entered the operating room in accordance with the principles of clean area, two buffer zones, and contaminated area, and wore biosafety level-3 (BSL-3) protective medical equipment. Orthopedic emergency patients were transferred to the negative pressure operating room (− 5 Pa or less) through a dedicated channel by staff wearing BSL-3 protective medical equipment. Continuous epidural anesthesia or combined spinal epidural anesthesia (CSE) was preferred to avoid infection from airway contact and the risk of exacerbating pulmonary complications by general anesthesia. For patients with critical condition, contraindications of epidural or CSE technique or failure of intrathecal anesthesia, general anesthesia should be selected. The minimally invasive, fast, and effective surgical methods should be used to abbreviate the surgical time. Patients were transferred to the isolation area of COVID-19 after operation.

### Statistical analysis

Data were statistically analyzed using GraphPad Prism version 8 (Graph-Pad Software Inc., San Diego, CA, USA) and SPSS statistical software (version 25 for Mac; IBM, New York, USA). Continuous variables are expressed as mean ± standard deviation (SD), and categorical variables are reported as number (*n*) and percentage (%).

## Results

### Epidemiologic characteristics

In the epidemic group, 82 orthopedic inpatients were enrolled in the present retrospective study, including 54 males (65.9%) and 28 females (34.1%) with an average age of 54.6 ± 20.2 years (range, 5.8–96 years). In the control group, there were 342 patients (male/female ratio, 193:149) with an average age of 48.6 ± 22.5 years. The age of the epidemic group was significantly older than that of the control group (*t* =  − 2.210, *P* = 0.028). For the epidemic group, 57 cases (69.5%) were traumatic fractures, and 25 cases (30.5%) were non-traumatic diseases, including malignant spinal tumor, spinal tuberculosis, degenerative spinal disease, and arthropathy. Among the 57 patients with fractures, 49 cases (86.0%) were caused by low-energy injuries, including flat ground injuries, sprains, and low-altitude (less than 1 m) fall injuries, and eight cases (14.0%) were caused by high-energy injuries, including three cases of car accident injuries, three cases of high-altitude (more than 3 m) fall injuries, and two case of the bruise injury caused by heavy object. For the control group, the proportion rates of traumatic factures (35.4%, 121/342) and low-energy injuries (37.2%, 45/121), which were significantly higher than that in the epidemic group (*χ*^2^ = 31.636, *P* < 0.001; *χ*^2^ = 36.988, *P* < 0.001) (Table 1).

**Table 1:** Clinical characteristics

Groups	The epidemic group	The control group	*P* value
Number	*N* = 82	*N* = 342	
Age, years; mean (SD)	54.6 (20.2)	48.6 (22.5)	*P* = 0.028
Gender, *n* (%)			*P* = 0.121
Male	54 (65.9%)	193 (56.4%)	
Female	28 (34.1%)	149 (43.6%)	
Injury priority, *n* (%)			*P* < 0.001
Priority A	6 (7.3%)	45 (13.2%)	
Priority B	8 (9.8%)	37 (10.8%)	
Priority C	68 (82.9%)	131 (38.3%)	
Priority D	0	89 (26.0%)	
Priority E	0	40 (11.7%)	
Type of diseases, *n* (%)			*P* < 0.001
Traumatic fractures	57 (69.5%)	121 (35.4%)	
Non-traumatic diseases	25 (30.5%)	221 (64.6%)	
Bone tumors	5 (6.1%)	27 (7.9%)	
Tuberculosis	4 (4.9%)	12 (3.5%)	
Degenerative spinal disease	12 (14.6%)	65 (19.0%)	
Arthropathy	4 (4.9%)	20 (5.8%)	
Others	0	97 (28.4%)	
Injury causes, *n* (%)			*P* < 0.001
Low-energy injuries	49 (86.0%)	45 (37.2%)	
High-energy injuries	8 (14.0%)	76 (62.8%)	
Fracture location, *n* (%)			*P* = 0.003
Elderly osteoporotic fractures (≥ 55 years)	28 (49.1%)	22 (18.2%)	
Limb fractures (≤ 55 years)	15 (26.3%)	63 (52.1%)	
Spinal cord injuries	7 (12.3%)	16 (13.2%)	
Multiple fractures	7 (12.3%)	20 (16.5%)	
Type of fracture, *n* (%)			*P* < 0.001
Closed fracture	53 (93.0%)	73 (60.3%)	
Open fracture	4 (7.0%)	48 (39.7%)	
Preoperative waiting time, mean (SD)	7.0 (2.6)	4.5 (2.1)	*P* < 0.001
Perioperative complications, *n* (%)	13 (12.2%)	12 (3.5%)	*P* < 0.001
Cardiovascular complications	2 (15.4%)	4 (33.3%)	
Venous thromboembolism	8 (61.5%)	3 (25.0%)	
Pneumonia	3 (23.1%)	5 (41.7%)	
Screening effect, *n* (%)			
Excluded COVID-19	80 (97.6%)		
Confirmed COVID-19	2 (2.4%)		
Nosocomial infection, *n* (%)	0		

### Comparison of admission and surgical indications

Among 82 inpatients in the epidemic group, the proportion of Priority A was 7.3% (6/82), followed by Priority B (8, 9.8%), and Priority C (68, 82.9%). For the control group, there were 45 Priority A, accounting for 13.2%, followed by Priority B (37, 10.8%), Priority C (131, 38.3%), Priority D (89, 26.0%), and Priority E (40, 11.7%). The orthopedic emergencies and expedited surgeries were indicative of admission and surgery in the epidemic group. The proportion of orthopedic emergency (17.1%, 14/82) and expedited surgery (82.9%, 68/82) in the epidemic group was significantly higher than that (24.0%, 82/342; 38.3%, 131/342) of the control group (*χ*^2^ = 60.383, *P* < 0.001) (Table 1).

### Surgical treatment

After confirming the exclusion of COVID-19 by the double-buffered process, the inpatients underwent orthopedic surgery. As of April 20, 2020, 71 patients had received surgical treatment and 11 patients were still waiting for surgery. The average time from admission to orthopedic surgery (7.0 ± 2.6 days) in the epidemic group was significantly longer than that (4.5 ± 2.1 days) of the control group (*t* =  − 9.223, *P* < 0.001) (Table 1).

### Perioperative complications

In the epidemic group, the postoperative complication rate was 12.2% (13/82), included cardiovascular complications (15.4%, 2/13), venous thromboembolism (61.5%, 8/13) and pneumonia (23.1%, 3/13). In the control group, the postoperative complication rate was 3.5% (12/342), included cardiovascular complications (33.3%, 4/12), venous thromboembolism (25.0%, 3/12) and pneumonia (41.7%, 5/12), which was lower than that in the epidemic group (*χ*^2^ = 18.170, *P* < 0.001) (Table 1).

### Evaluation of nosocomial infection

A total of 33 orthopedic surgeons and 34 nurses in two orthopedic wards were involved in the process of orthopedic diagnosis and treatment. All medical staff used BSL-3 protective equipment in the outpatient clinic, comprehensive buffer ward, and orthopedic ward buffer room. Thus far, no nosocomial infections of COVID-19 occurred between doctors and patients, as determined by quantitative real-time polymerase chain reaction testing and chest CT results. Among the hospitalized patients, two cases of asymptomatic infection were screened out and transferred to the designated hospital for COVID-19 (Table 1).

## Discussion

The current study presents the clinical data of patients in orthopedic surgery during the COVID-19 outbreak and the corresponding period last year in Wuhan, China. Eighty-two inpatients underwent preliminary emergency or outpatient screening and repeated screening in comprehensive and orthopedic buffer wards. The findings indicate that orthopedic inpatients have unique epidemiological characteristics; in particular, traumatic factures mainly presented with low-energy fractures. Our experience of using a double-buffered diagnosis and treatment mode provides a reliable reference for the future treatment of orthopedic patients.

The diagnosis and treatment of orthopedic patients was significantly affected by the outbreak of COVID-19, which changed the mode of orthopedic clinical practice. The guiding principles of clinical orthopedic work include the following aspects: (1) the urgency of the patient’s condition; (2) the protection of patients and medical staff, and (3) the reasonable use of medical resources. In our hospital, orthopedic surgery and ward management changes were adjusted accordingly on the basis of these principles. Patients who required emergency or early orthopedic surgery intervention were admitted to the orthopedic hospital as soon as possible. During the COVID-19 epidemic, patients with fractures due to high-energy injuries, such as transportation and engineering construction were relatively rare because of the strict control in Wuhan, China. Accidental fall injuries during home isolation activities are common, and the majority of these fracture patients were relatively stable, especially among the elderly [10]. Our results showed that the age of epidemic period was significantly older than that of non-epidemic period. Orthopedic patients in need of emergency treatment, including open fractures, osteofascial compartment syndrome, blood vessels and nerves injury, and large area avulsion skin injuries, were significantly reduced because of strict city control measures [11]. During the epidemic period, the orthopedic emergencies and expedited surgeries were indicative of admission and surgery, while the elective surgery was 37.7% during the non-epidemic period. In this work, several patients with closed fractures caused by low-energy injuries needed to be treated, and most of these treatments were limited-time surgeries. Limited-time operations performed within 1–2 weeks will not affect their clinical effect. Considering the potential risk of SARS-CoV-2 transmission and spread in the hospital, patients who need elective surgery could choose outpatient prescriptions and consider temporary pain-alleviating measures, arthroscopy (shoulders, knees, and ankles), knee and hip arthroplasty, spinal deformity corrections, and implant removals, in their care [12]. Elective surgical cases were postponed, which could greatly reduce the workload of orthopedic surgeons and avoid a major drain of healthcare resources during an epidemic [13]. Orthopedists have also been advised to prolong the duration between non-urgent follow-ups to avoid patient overcrowding in hospitals.

During the COVID-19 epidemic in Wuhan, osteoporotic fractures in the elderly, especially hip fractures and vertebral fractures, were common. Among the cases included in this study, 28 (49.1% of the fracture cases) had osteoporotic fractures. The patients were mostly in poor physical condition and presented with a combination of various diseases, including hypertension, heart disease, and diabetes [14]. These patients were recommended for hospitalization for early surgical treatment, which can reduce various complications caused by long-term bed rest, including lung infections, urinary system infections, deep vein thrombosis, and bedsores [15, 16]. In the present study, two cases who fell from a vertical height of over 3 m presented multiple fractures. These two patients had an average age of 24.5 years and a history of previous mental illness. During an epidemic, patients may feel a sense of uncertainty and helplessness, resulting in different levels of psychological/behavioral stress responses, psychological problems, and even mental disorders [17]. Therefore, counseling and health management are recommended for this group of patients. Patients with bone tumors and tuberculosis, especially vertebral tumors and tuberculosis, have strong requests for hospitalization [18]. Pain symptoms and paralysis due to deterioration of the lesion may become unbearable, and the urgency of medical treatment is only slightly lower than that of trauma fracture. In the present study, eight patients hospitalized with vertebral tumors and tuberculosis delayed diagnosis and treatment until the most severe period of the epidemic. When serious complications occurred, including unbearable pain and paralysis caused by spinal cord compression, patients chose to be hospitalized in time. The diagnosis and treatment of these patients’ diseases were delayed because of COVID-19, which may eventually lead to poor prognosis.

There is conflicting evidence regarding the relationship between surgical delay and clinical outcome in patients with traumatic fractures [19]. Most trauma patients were afraid of going out or did not realize the severity of their traumatic condition. They chose to stay at home for temporary observation. In addition, the 2-day COVID-19 RNA and antibody detection assays were the necessary prerequisite for allowing patients to enter the buffer ward. These factors could explain the patients’ delay from admission to surgery. The increase in waiting time was closely related to the increased risk of perioperative complications in surgical patients, especially those with traumatic fractures. Common complications during the perioperative period mainly included cardiovascular complications, venous thromboembolism and pneumonia, which were related to the patients’ limb dysfunction, reduced activity and long-term bed rest [20]. In the epidemic group, the postoperative complication rate was 12.2%, which was higher than that in the non-epidemic period.

National recommendations and local infection control guidelines are tailored based on the availability of medical resources and the severity of the epidemic. The staff in Department of Bone & Joint Surgery, Peking University Shenzhen Hospital had benefited from the strict flowcharts, smart robot, and protection equipment during the perioperative managements for orthopedic patients. With the help of the strict flow charts and smart equipment, post-operation outcomes of the patients revealed that the rates of the complications and re-operation had been reduced significantly [21]. The Department of Orthopedics and Orthopedic Oncology, University of Padova shared their experience. They made changes by medical direction to reallocate resources to COVID-19 patients, and a decrease in the number of beds and surgical activity was stabilized [22]. Former researchers have provided good prevention and control facilities for the benefits of prevention and control. These strategies are beneficial to both the orthopedic patients and the medical staff.

Preventive and control measures should be formulated in a targeted manner according to the clinical characteristics of inpatients during an epidemic to standardize procedures for patient visits and hospitalization, treat patients rationally, and reduce the incidence of nosocomial infection. Patients may either be carriers of COVID-19 or asymptomatic cases. Thus, establishing strategies to prevent and control COVID-19 while implementing good orthopedic treatment and avoid SARS-CoV-2 spreads between doctors and patients is of great important. The workflow of the double-buffered diagnosis and treatment mode was implemented to standardize the treatment of orthopedic patients. After screening through the emergency triage process, patients undergo clinical medical observation in the surgical comprehensive buffer ward, receive COVID-19-related examinations, and perform primary buffering. During the screening process, confirmed COVID-19 patients are immediately transferred to the isolation ward to prevent patients missed during outpatient screening from entering the orthopedic ward. In our double-buffer mode, patients in the comprehensive buffer zone were transferred to the orthopedic buffer protection room after a clinical observation period of 2–3 days. Then, they undergo a second buffer to improve the operating efficiency of the whole buffer ward and ensure a safe treatment environment for doctors and patients. The entire COVID-19 screening buffer transition period is approximately 4–6 days, consistent with the timing of surgical treatment for most patients with trauma fractures; this period does not affect the patient’s condition and orthopedic treatment. The average waiting time of patients before surgery in this study was 7.0 days, which is relatively longer than that in the non-epidemic period. After strict emergency triage and double-buffering procedures, the patients are transferred to the orthopedic safe patient ward to ensure the safety of patients and orthopedic medical staff. For patients with life-threatening or multiple injuries, when emergency surgery is required, level III protection and multidisciplinary collaboration should be adopted in the negative pressure operation without confirmation of whether the patient is suspected or confirmed, and screening can be conducted in a separate isolated environment postoperatively. Each link has the risk of infection, which needs to be implemented in accordance with the requirements of level III protection to reduce the risk of nosocomial infection. Thus, our mode lays a good foundation for the safe diagnosis and treatment of orthopedic patients. We believe that our double-buffer mode can solve difficulties related to epidemic prevention and control during the treatment of orthopedic patients.

Surgeons must provide utmost care to patients in the preoperative, intraoperative, and postoperative settings to minimize the risk of nosocomial spread [18]. The risks and benefits of surgical management should be reasonably considered for each patient. In this study, all patients were contacted 1 day before the operation and checked for respiratory symptoms, risk factors, or recent travel history (within 14 days) that may put them at risk of COVID-19. Operative personnel should be minimized, and surgical times should be kept as short as possible [23]. Doctors may be segregated into an inpatient team that attends to patients in wards, operates, and provides on-call service and an outpatient team who is responsible for special orthopedic outpatient services. Hospitals should be in lockdown with no visitors allowed. The emergence of such a crisis provides a timely opportunity for clinicians to reflect and evaluate the use of novel technologies in the workplace [24]. For example, the traditional work mode of ward round and shift handover could be adjusted, and a modern network technology could be adopted to transmit information. Clinical affairs, such as hospital consultation, difficult case discussion, and disease communication, could be conducted through WeChat, QQ and other ways. Furthermore, a simple, convenient, and efficient ward round system and management mode could be implemented to reduce medical staff gathering and doctor–patient contact.

The role of orthopedic surgeons in alleviating the COVID-19 crisis is certainly an important one. Even when reviewing low-risk elective patients, doctors should be vigilant, advocate good hygiene, and maintain an open mind when adopting novel workplace techniques. The shortcomings of this study are that the current diagnosis and treatment mode is fairly new, and the diagnosis and treatment process requires further adjustment according to the different stages of epidemic prevention and control. However, we believe that the proposed diagnosis and treatment mode will eventually be fine-tuned to provide a reliable and accurate reference for clinical work during an epidemic.

Several limitations to our work should be discussed. Firstly, the inherent limitations of retrospective design may compromise the accuracy of data collection. Secondly, due to the relatively small number of patients included and the data from a single-center study, selection bias was a concern. Our findings should be confirmed in a large-scale randomized trial in the future.

## Conclusion

The current study presented the epidemiological characteristics of patients between COVID-19 epidemic and non-epidemic periods. Orthopedic inpatients had unique epidemiological characteristics during the COVID-19 outbreak in Wuhan, China; in particular, traumatic factures mainly presented low-energy fractures. While the double-buffered strategy could extending the patients’ preoperative waiting time, the risks of nosocomial spread can be effectively minimized. This study also suggests that under such a preoperative screening mode, doctors should pay more attention to perioperative management in order to prevent or reduce complications.

## Data Availability

The datasets used and/or analyzed during the current study are available from the corresponding author on reasonable request.
